# Supplementary material for: Realistic in silico generation and augmentation of single-cell RNA-seq data using generative adversarial networks
Source: Nat Commun. 2020 Jan 9;11:166. doi: 10.1038/s41467-019-14018-z (PMC6952370; doi:10.1038/s41467-019-14018-z)
Supplement: Supplementary file 2 — Supplementary Information [file 41467_2019_14018_MOESM2_ESM.pdf]

Supplementary Information

Realistic *in silico* generation and augmentation of single-cell RNA-seq data using Generative Adversarial Networks

Marouf *et al*.

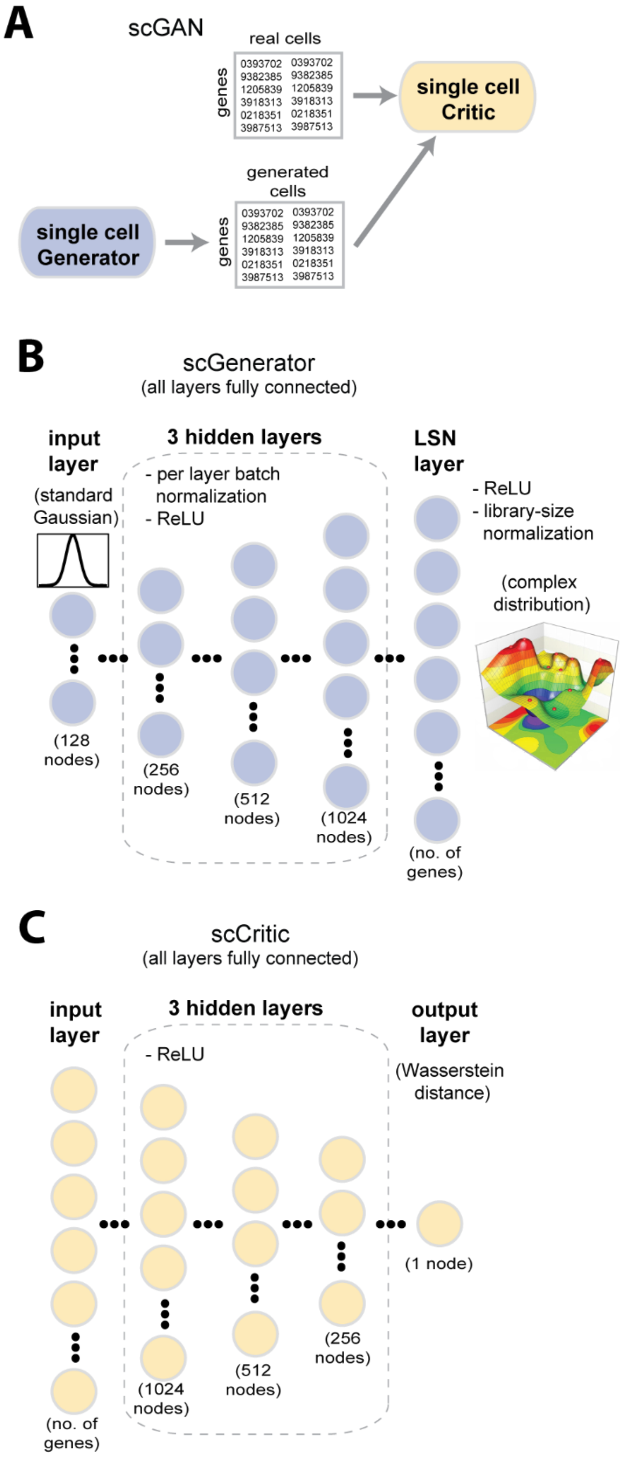

Supplementary Figure 1 *Schematic representation of the scGAN.* A: High-level architecture of the scGAN. B: Architecture of the generator network. The generator consists of a Fully-Connected network with three hidden layers of growing size, each featuring Batch Normalization and ReLU activation, and a Library-Size Normalization output layer. The inputs are realizations of standard Gaussian noise and the outputs are single cell expression levels

that resemble the training cells. C: Architecture of the critic network. The critic consists of a Fully-Connected network with three hidden layers of decreasing size with ReLU activation.

Dataset	Source	Organism	Tissue	Cell number	Gene number	Louvain clusters
PBMC	10x Genomics	*Homo sapiens*	Blood	68,579	17,789	10
Brain small	10x Genomics	*Mus musculus*	Brain	20,000	17,970	8
Brain large	10x Genomics	*Mus musculus*	Brain	1,306,127	22,788	13
Bone Marrow	GEO	*Mus musculus*	Brain	2,730	12,443	7
Zeisel	GEO	*Mus musculus*	Brain	3,005	18,738	6

Supplementary Table 1 *scRNA-seq datasets used.* Description of the datasets used throughout the manuscripts, displaying the species and tissue of origin, the number of cells and genes expressed, and the number of clusters inferred with the Louvain method.

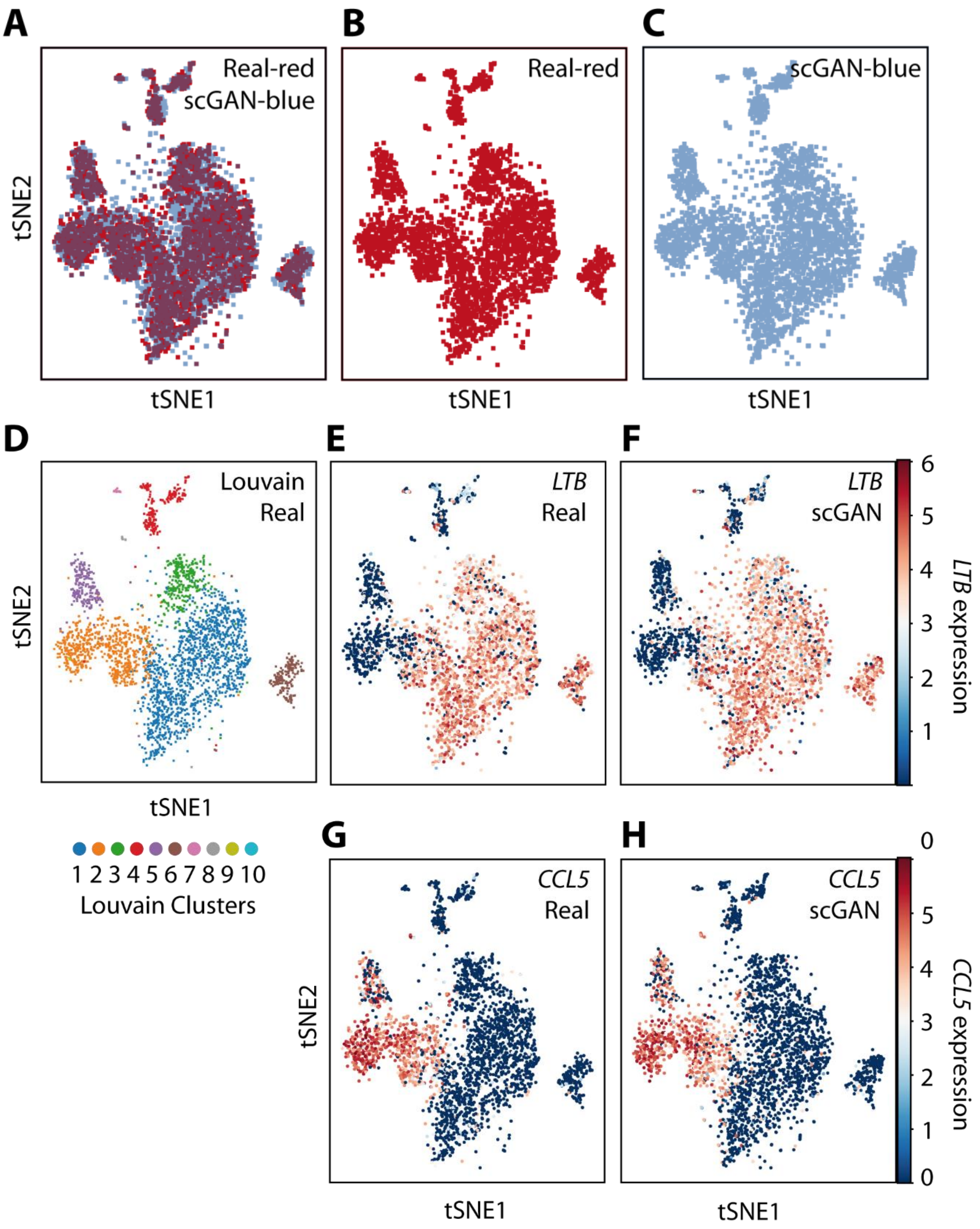

Supplementary Figure 2 *t-SNE visualizations of real and scGAN generated PBMC cells.* A-C: Real cells are shown in red (panels A and B) and generated cells in blue (panels A and C). D: Real cells are shown with their Louvain clustering. E-F: LTB gene expression for real (panel E) and scGAN generated (panel F) cells. G-H: CCL5 gene expression for real (panel G) and scGAN generated (panel H) cells.

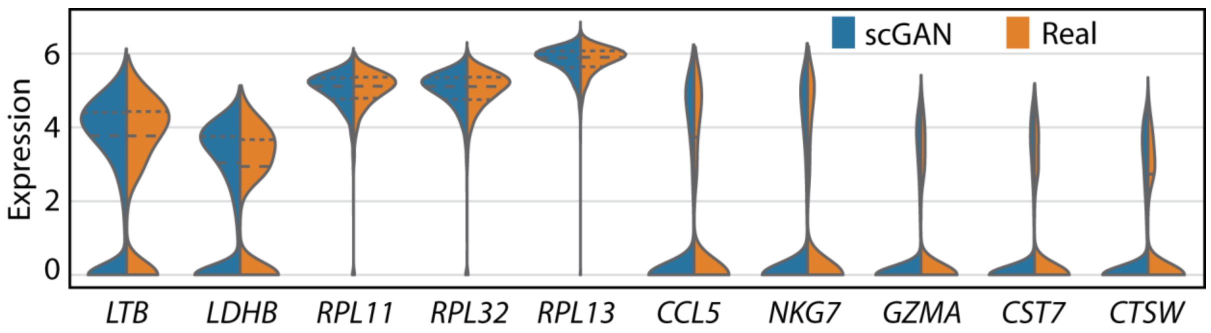

Supplementary Figure 3 *Expression of ten marker genes for real and generated cells.* Split violin plots of the logarithmic expression distribution of the top five marker genes of cluster 1 (LTB, LDHB, RPL11, RPL32, RPL13) and cluster 2 (CCL5, NKG7, GZMA, CST7, CTSW). Blue corresponds to scGAN generated cells, orange to real data.

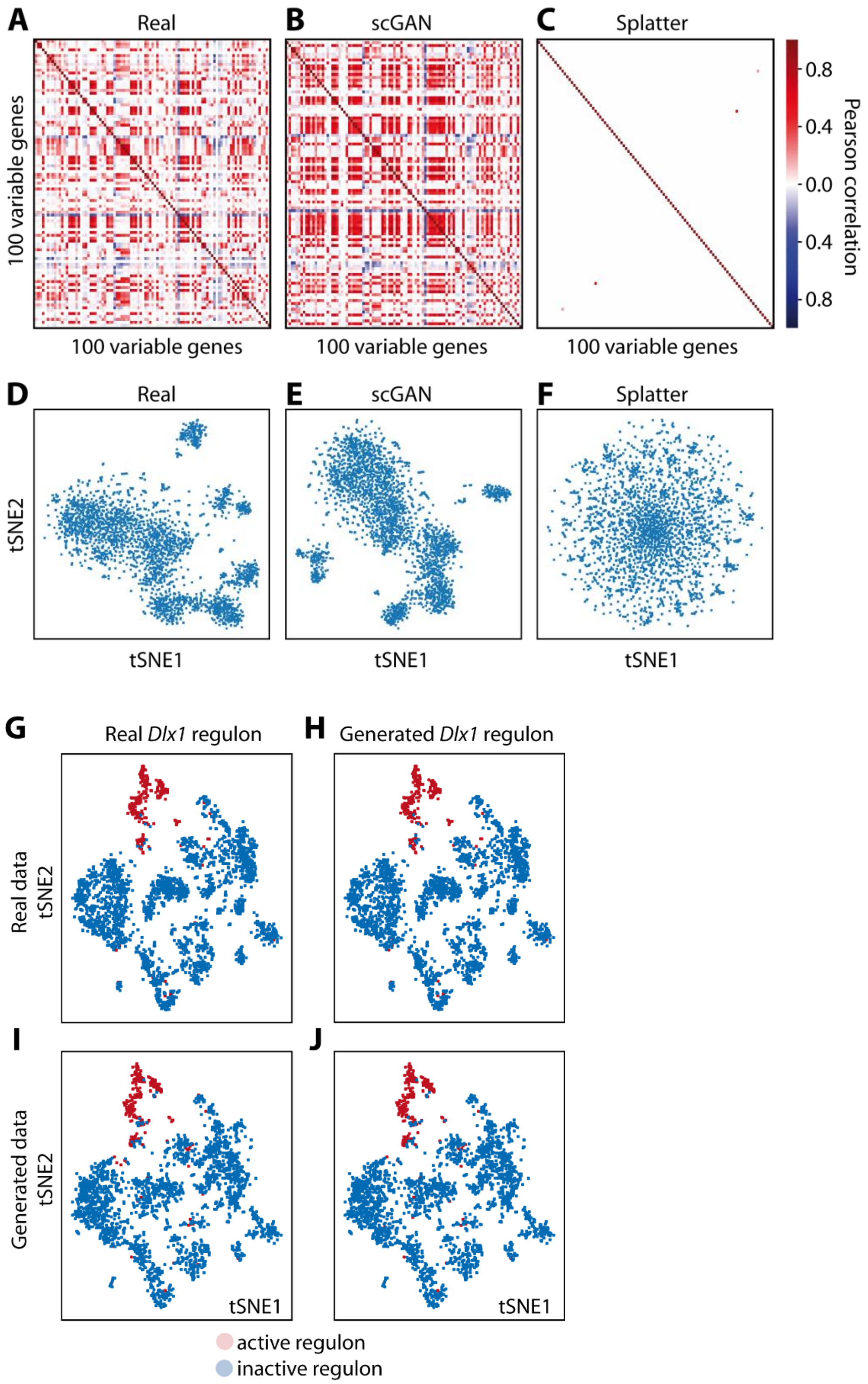

Supplementary Figure 4 *Comparison of Real, scGAN-, and Splatter-generated gene correlations and cell clustering.* A-C: Pearson correlation of the 100 most highly variable genes for Real (panel A), scGAN-generated (panel B), and Splatter-generated (panel C) data. It should be noted that the 100 most highly variable genes were calculated for Real, scGAN, and

Splatter data separately, as Splatter does not keep the gene information of the original data. Model parameters were learned using the PBMC data. D-F: t-SNE visualizations of Real (panel D), scGAN-generated (panel E), and Splatter-generated (panel F) cells. It is to be noted that different t-SNE embeddings were used for each t-SNE plot since Splatter does not keep the gene information of the original data. Models were learned using the PBMC data. G-J: t-SNE visualizations of Real (test) (panels G and H) and scGAN-generated (panels I an J). Binary activation of the regulon is displayed (cells where the regulon is active in red, and inactive in blue) for the Dlx1 regulon inferred with SCENIC from the Real cells (panels G and I) and from the scGAN-generated cells (panels H and J).

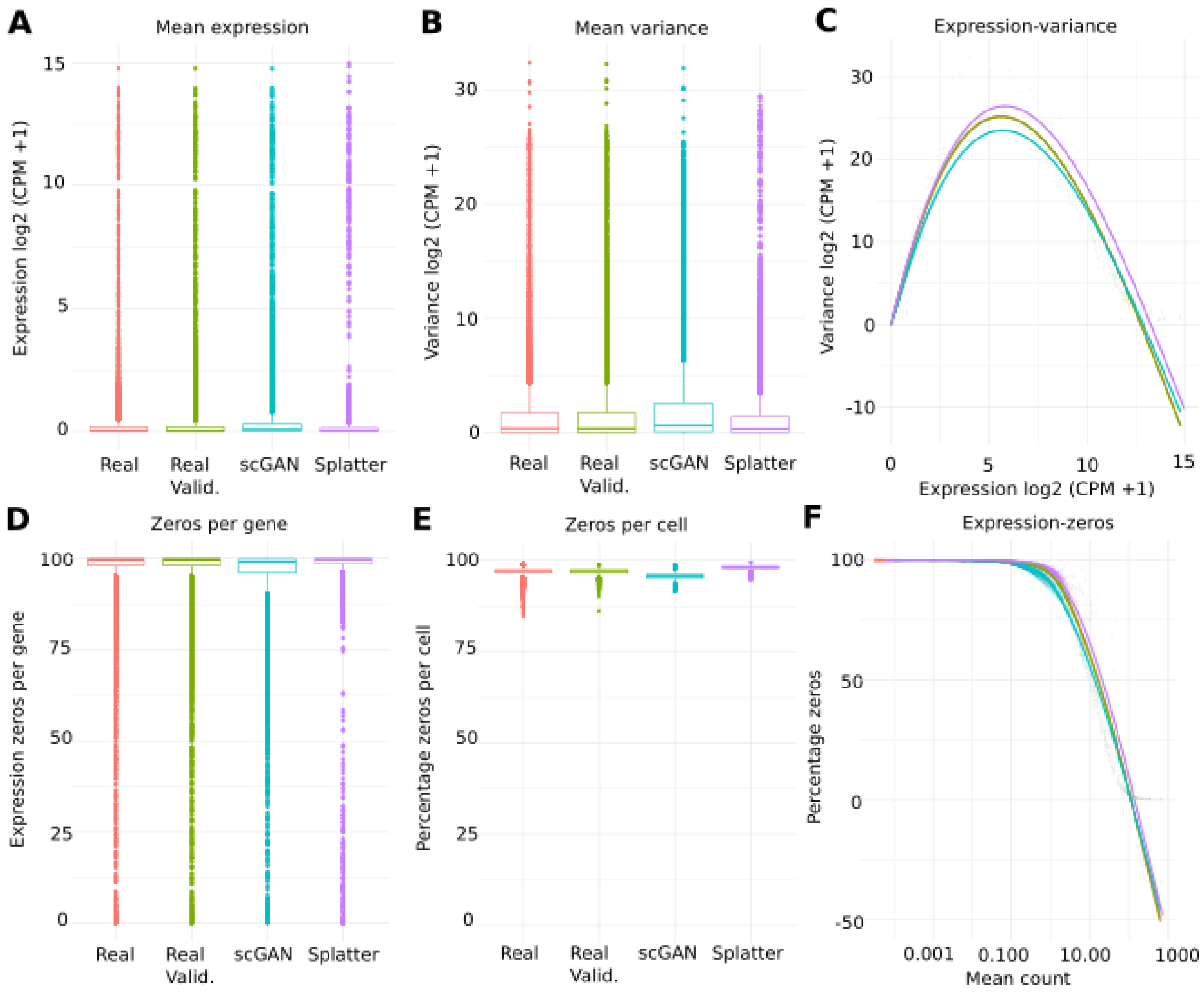

Supplementary Figure 5 *Basic statistical evaluation of the real and scGAN and Splatter generated data.* A: Box plot of the mean expression per cell (in logarithm of Counts Per Million) in real training (red), real test (green), scGAN generated (turquoise), and Splatter generated (purple) cells. B: Box plot of the mean variance per cell. C: Mean variance (y-axis) against the per cell mean expression (x-axis). D: Box plot of the percentage of zero expression values per gene. E: Box plot of the percentage of zero expression values per cell. F: Mean count (x-axis) against the percentage of zero expressed genes per cell (y-axis).

	Real (training)	scGAN	Splatter	SUGAR
MMD score	0.037	0.872	129.52	59.45

Supplementary Table 2 MMD statistics computed between the real (test) cells and the cells generated by the different models (scGAN,Splatter and SUGAR). The MMD score of real (training) cells was used as a positive control.

Cluster	Real (training)	scGAN	cscGAN
All	0.080	0.547	0.674
2	0.037	n/a	0.286
6	0.129	n/a	0.238

Supplementary Table 3 MMD statistics computed between the real (test) cells and the cells generated by the different models (scGAN, cscGAN). The MMD score of real (training) cells was used as a positive control. Note that the scGAN cannot directly generate cluster-specific cells so that it is not possible to obtain the corresponding MMD scores.

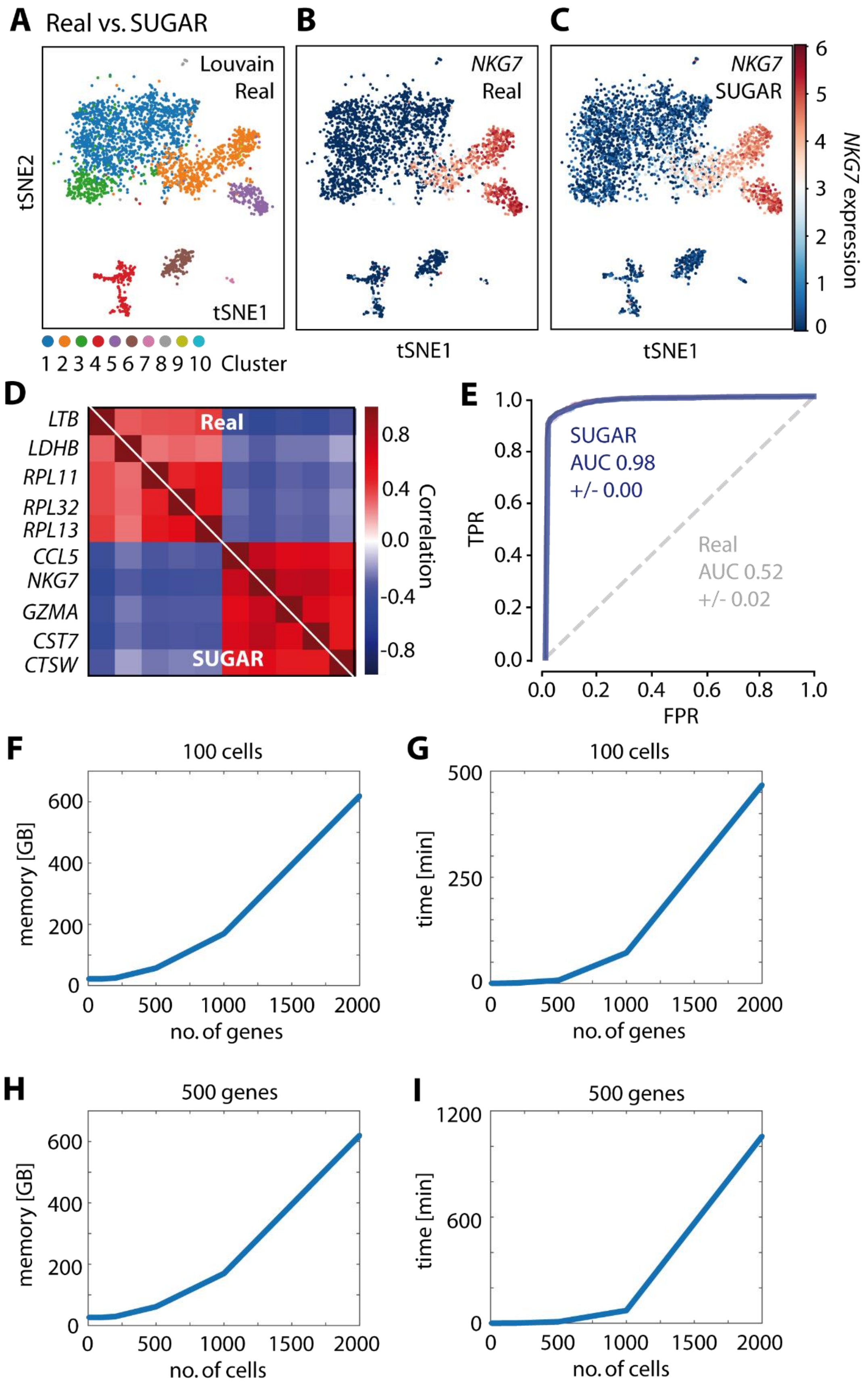

Supplementary Figure 6 *Evaluation of SUGAR generated PBMC cells.* A-C: t-SNE visualization of the clustered real cells (panel 1) and the NKG7 gene expression in real (panel

B) and SUGAR generated (panel C) cells. D: Pearson correlation of marker genes for the SUGAR generated (bottom left) and the real (upper right) data. E: Cross validation ROC curve of an RF classifying real and generated cells (SUGAR in blue, chance-level in gray). F-I: Runtime (panels F and H) and memory usage (panels G and I) of SUGAR for increasing number of genes (panels F and G) and cells (panels H and I), using kernel estimation and density equalization to generate 10.000 cells. Experiments were performed on a Dell Power Edge R940 server with 128 x 2.6 GHz Intel Xeon threads and 1.47 TByte of RAM.

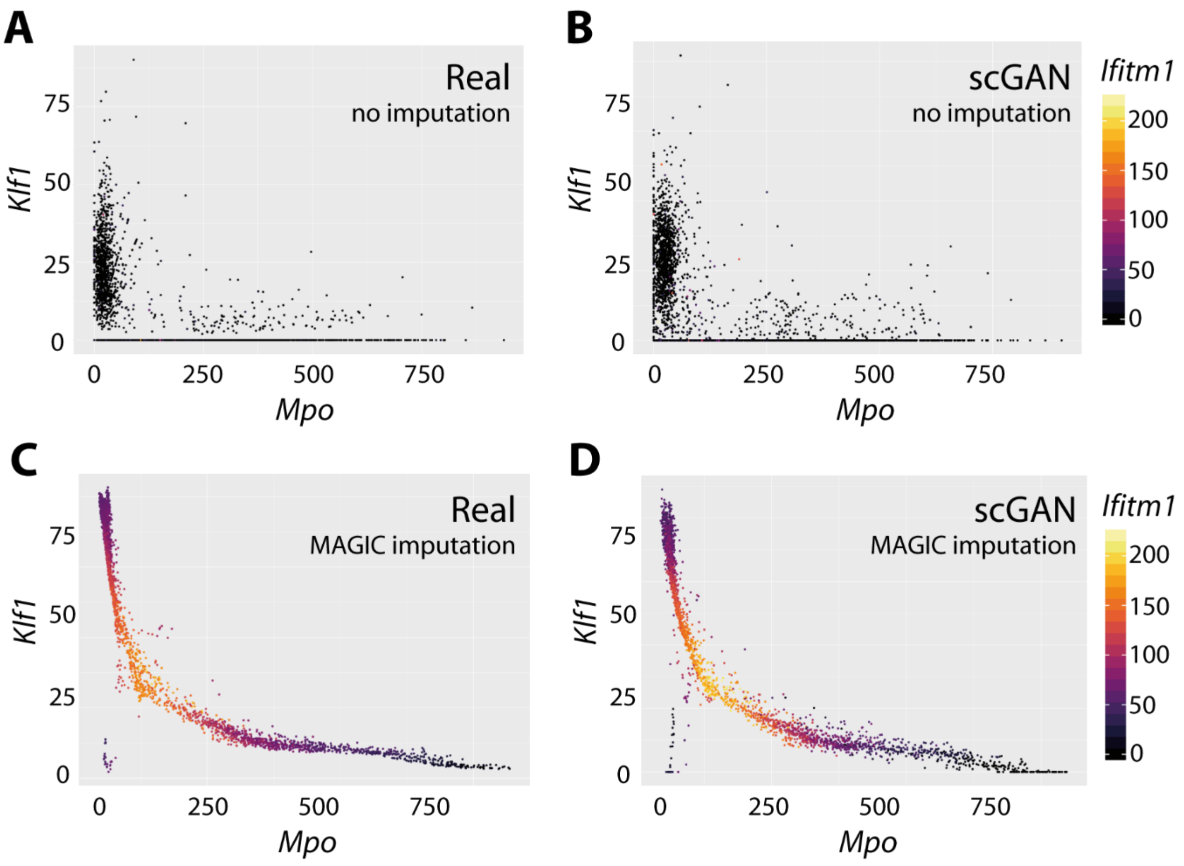

Supplementary Figure 7 *scGAN can model MAGIC imputed scRNA-seq data.* scGAN models have been trained on scRNA-seq data without (A, B) and with prior gene expression imputation using MAGIC (C, D). Real (A) and scGAN (B) generated data show little correlations between *Mpo, Klf1*, and *Ifitm1* expression. The scGAN models the input data realistically and does not change or impute gene expression values. C: scRNA-seq data that has been imputed with MAGIC shows a strong non-linear correlation between *Mpo, Klf1*, and *Ifitm1*. D: The correlation between *Mpo, Klf1*, and *Ifitm1* is conserved in scGAN generated data that was imputed with MAGIC prior to training.

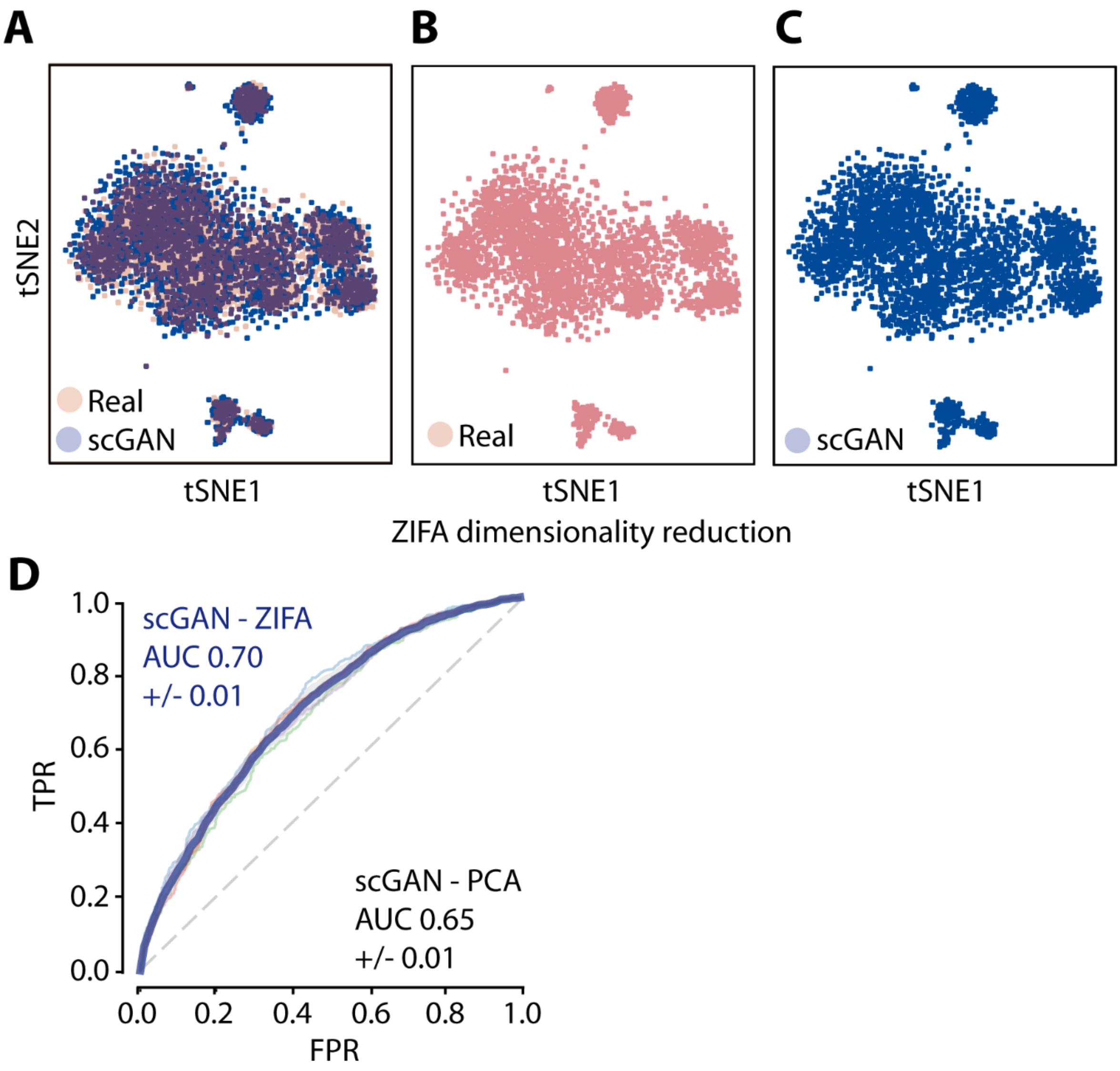

Supplementary Figure 8 Evaluation of the scGAN generation of PBMC cells using ZIFA dimensionality reduction. A-C: ZIFA-based t-SNE visualization of real cells (red, panels A and B), generated cells (blue, panels A and C). D: Cross-validation ROC curve of a ZIFA-based RF classifying real from scGAN generated cells (scGAN in blue, chance-level in gray). The AUC obtained with a PCA-based RF classification is recalled in the bottom right corner.

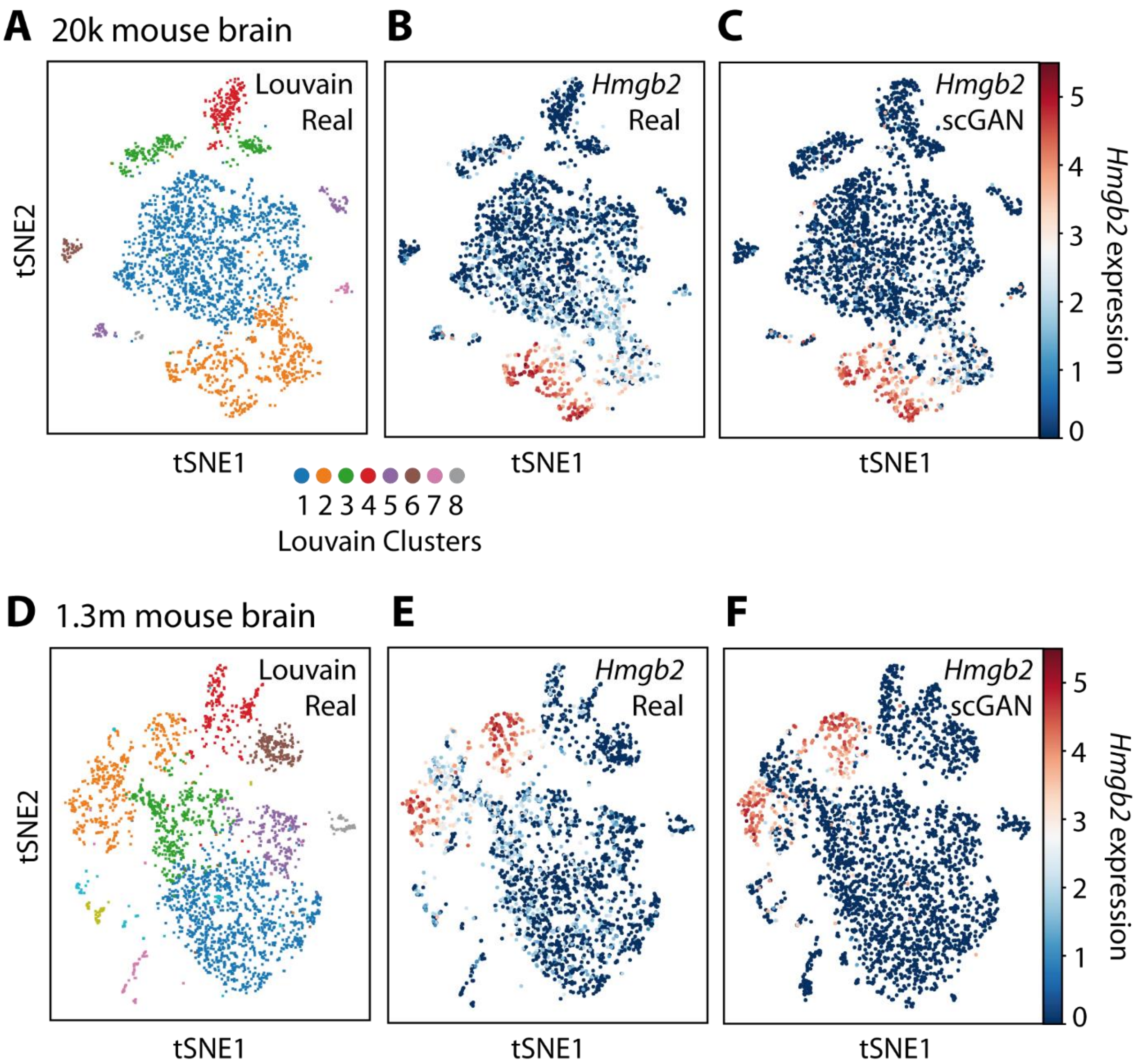

Supplementary Figure 9 *Evaluation of the scGAN simulated Brain Small and Brain Large cells.* A-C: t-SNE visualization of Louvain-clustered real cells (panel A) and the *Hmgb2* gene expression in real (panel B) and scGAN generated (panel C) cells for the Brain Small dataset (20k mouse brain). D-F: t-SNE visualization of Louvain-clustered real cells (panel D) and the *Hmgb2* gene expression in real (panel E) and scGAN generated (panel F) cells for the Brain Large dataset (1.3 million mouse brain).

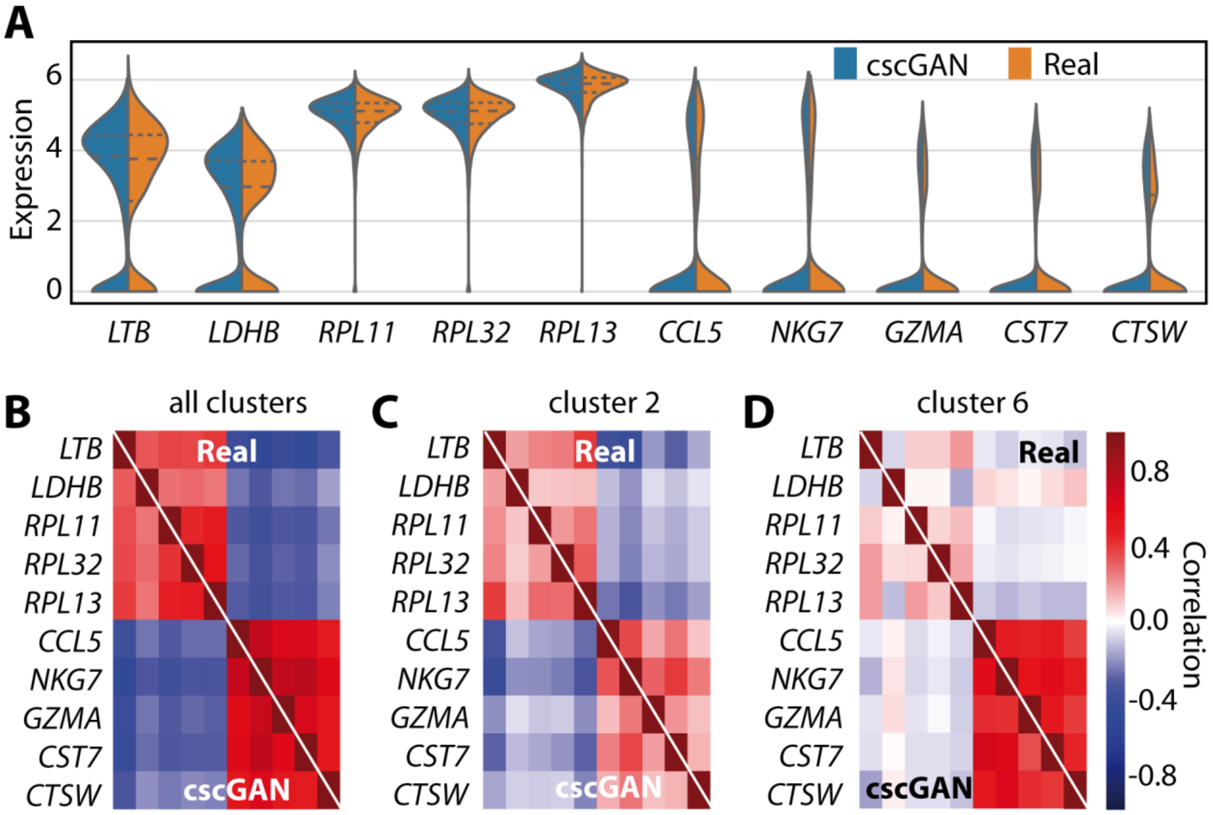

Supplementary Figure 10 *Expression and correlation of ten marker genes for real and conditionally generated PBMC cells.* A: Split violin plots of the distribution of the top five marker genes of cluster 1 (*LTB*, *LDHB*, *RPL11*, *RPL32*, *RPL13*) and cluster 2 (*CCL5*, *NKG7*, *GZMA*, *CST7*, *CTSW*). Blue corresponds to cscGAN generated cells, orange to real data. B-D: Pearson correlation of marker genes for the scGAN generated (bottom left) and the real (upper right) data for (B) all cells, (C) cluster 2, and (D) cluster 6 cells.

Cluster	Projection GAN	ACGAN	Real
1	0.65 ± 0.02	0.76 ± 0.02	0.55 ± 0.01
2	0.62 ± 0.03	0.69 ± 0.01	0.51 ± 0.03
3	0.61 ± 0.05	0.78 ± 0.01	0.55 ± 0.06
4	0.60 ± 0.07	0.98 ± 0.01	0.51 ± 0.03
5	0.63 ± 0.07	0.66 ± 0.08	0.44 ± 0.03
6	0.55 ± 0.03	0.98 ± 0.02	0.44 ± 0.04
All	0.63 ± 0.01	0.69 ± 0.01	0.53 ± 0.01

Supplementary Table 4 *Overview of RF classification performance discriminating real from cscGAN generated cells.* Cross-validation area under the ROC curve (AUC) of RFs classifying between real and cscGAN generated cells using a projection (Projection GAN) or an ACGAN critic. As control, we also show the classification performance on real training data, which should have chance-level performance (Real). The first six rows correspond to the classification performance for specific clusters (clusters 1-6, other clusters are too small for proper classification), while the last row highlights the classification performance across all clusters (clusters 1-10). For each cell of this table, the left value represents the average AUC across the five folds of the cross-validation. The right value corresponds to the standard deviation.

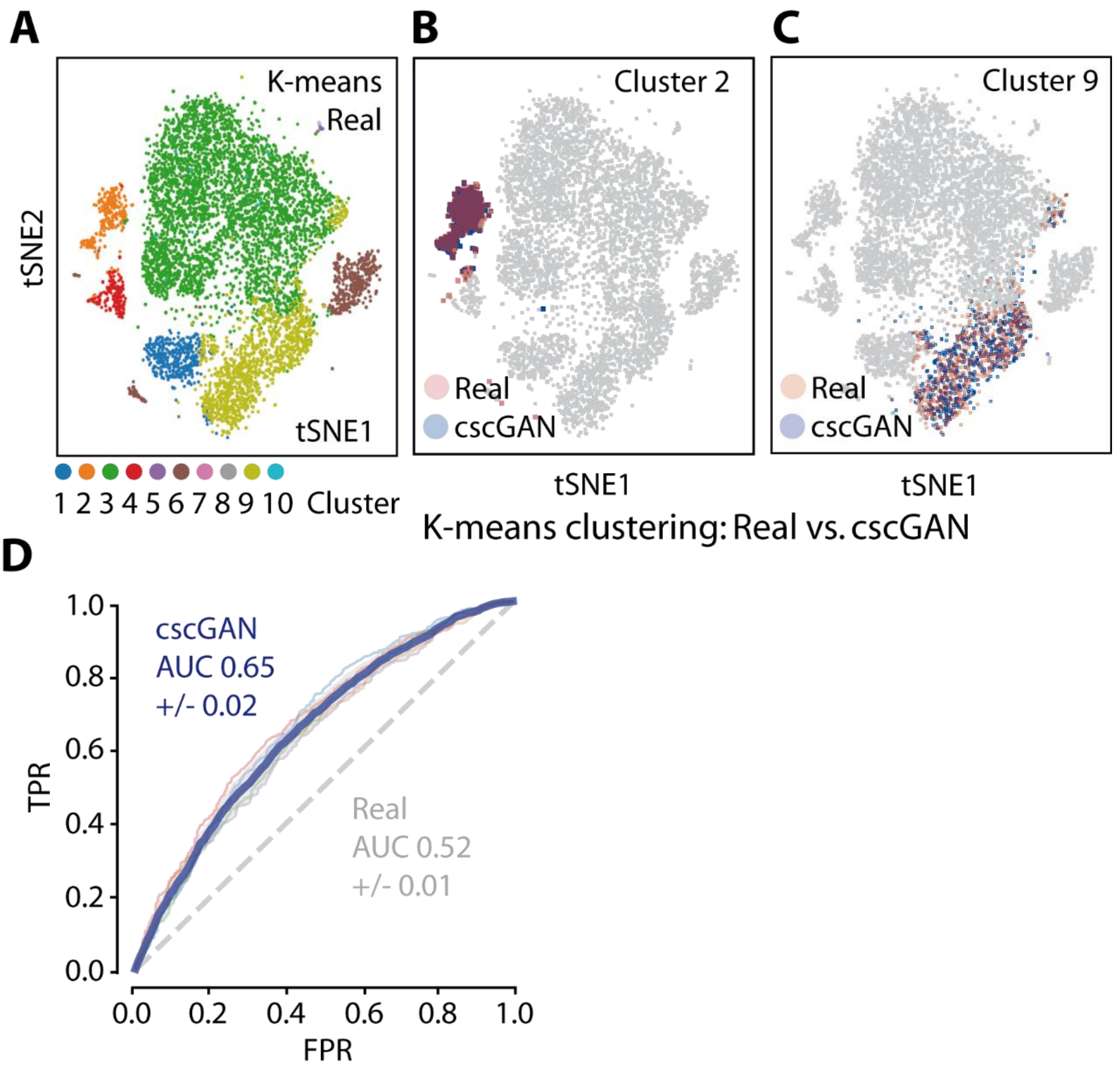

Supplementary Figure 11 *Evaluation of the cscGAN trained on k-means clustered data.* A: t-SNE visualization of K-means clusters of real cells. B: t-SNE visualization of cluster 2 real cells (red), cluster 2 generated cells (blue), and other real cells (grey). C: t-SNE visualization of cluster 9 real cells (red), cluster 9 generated cells (blue), and other real cells (grey). D: Cross-validation ROC curve of an RF classifying cluster 2 real from cscGAN generated cells (cscGAN in blue, chance-level in gray).

Cluster	Projection GAN	Real
1	0.54 ± 0.05	0.46 ± 0.05
2	0.60 ± 0.03	0.53 ± 0.06
3	0.67 ± 0.01	0.54 ± 0.02
4	0.59 ± 0.07	0.55 ± 0.09
5	0.50 ± 0.04	0.47 ± 0.02
6	0.62 ± 0.03	0.52 ± 0.02
All	0.64 ± 0.02	0.52 ± 0.01

Supplementary Table 5 *Overview of RF classification performance discriminating real from cscGAN generated cells, using K-means cluster indices for conditioning.* Cross-validation area under the ROC curve (AUC) of RFs classifying between real and cscGAN. As control we also show the classification performance on real training data, which should have chance-level performance (Real). The first six rows correspond to the classification performance for specific clusters (clusters 1-6 other clusters are too small for proper classification), while the last row highlights the classification performance across all clusters (clusters 1-10). For each cell of this table, the left value represents the average AUC across the five folds of the cross-validation. The right value corresponds to the standard deviation.

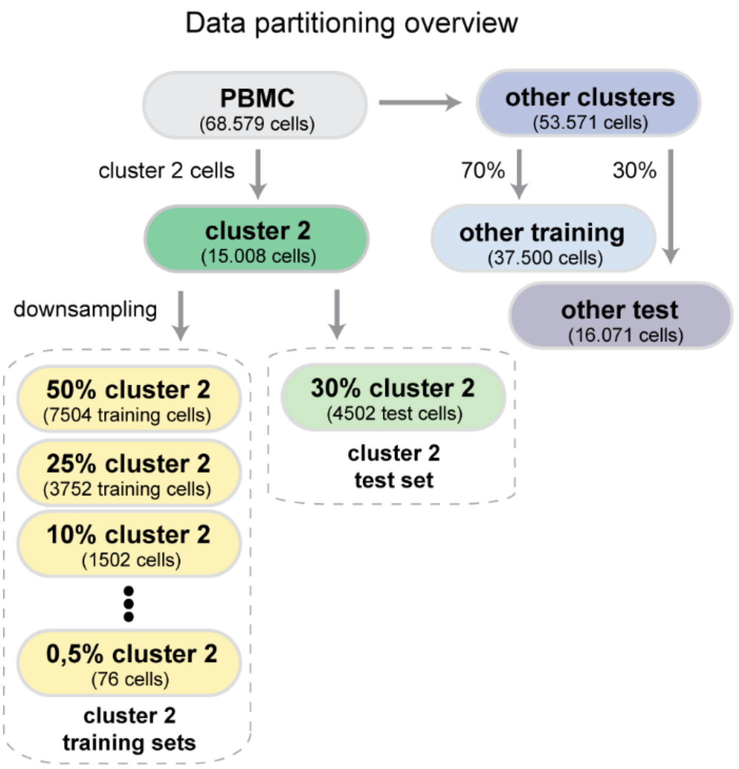

Supplementary Figure 12 *Overview of the PBMC data partitioning for downsampling and augmentation experiments.* Cells from the cluster 2 population (dark green) are split into a cluster 2 training set that is downsampled into eight datasets with different cell numbers (yellow), and a test set of 30% of all cluster 2 cells (light green). Cells from other clusters are split into a training set (other training, light blue, 70% of other clusters set) and a test set (other test, dark blue, 30% of other clusters set).

Percentage	50%	25%	10%	5%	3%	2%	1%	0.5%
Training cells	7504	3752	1501	751	451	301	151	76

Supplementary Table 6 *Number of cluster 2 cells used for all eight levels of downsampling.*

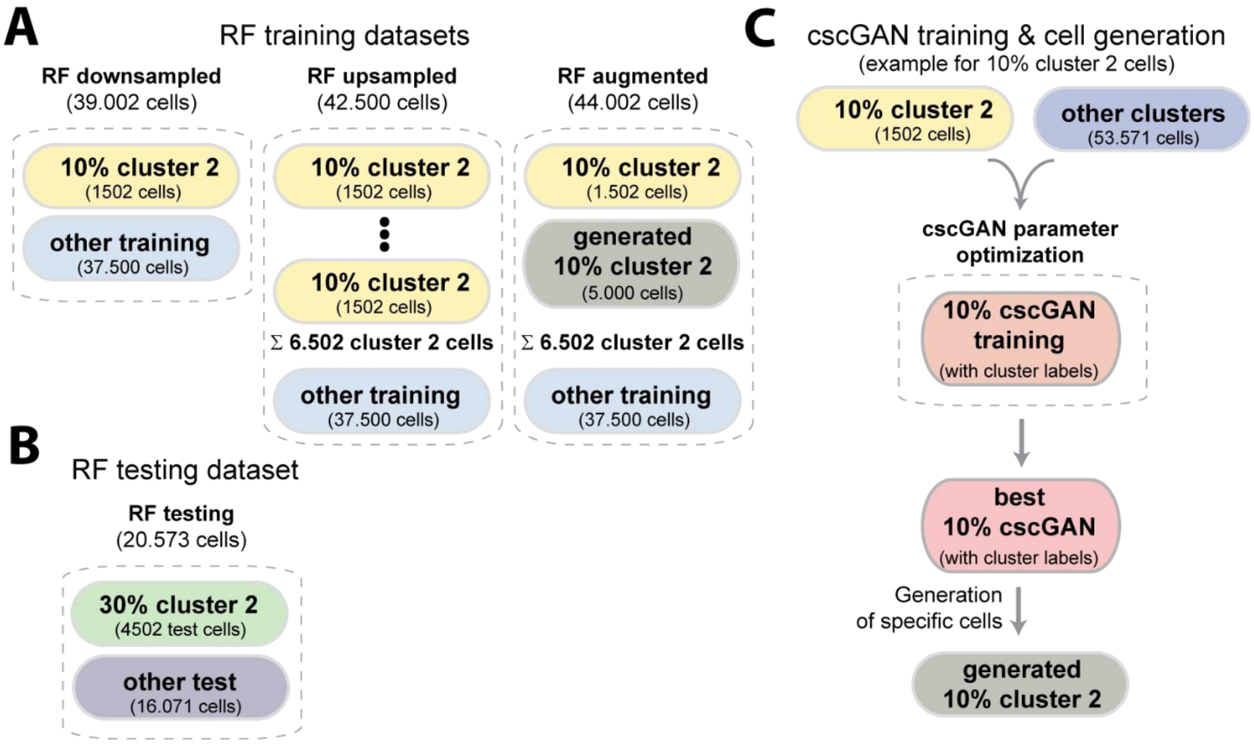

Supplementary Figure 13 *Schematic representation of the datasets used for classification and cell generation.* A: RF training was conducted on three different datasets for each percentage of downsampling of cluster 2 cells (as an example we use 10% in the figure). The RF downsampled dataset consists of the 10% cluster 2 set (yellow). The RF upsampled dataset contains 6,502 cluster 2 cells sampled with replacement from the 10% cluster 2 set. The RF augmented dataset contains the 10% cluster 2 set, and 5,000 cells generated using the generated 10% cluster 2 set (grey, see also panel C). In addition, all three datasets contain the other training set (light blue). B: RF testing was conducted on the 30% cluster 2 test set (green) and the other test set (dark blue). C: For data augmentation (see RF augmented) we generate cluster 2 cells using a cscGAN. The cscGAN is trained on the 10% cluster 2 set (yellow) and the other clusters set (blue), yielding a generated 10% cluster 2 set (gray).

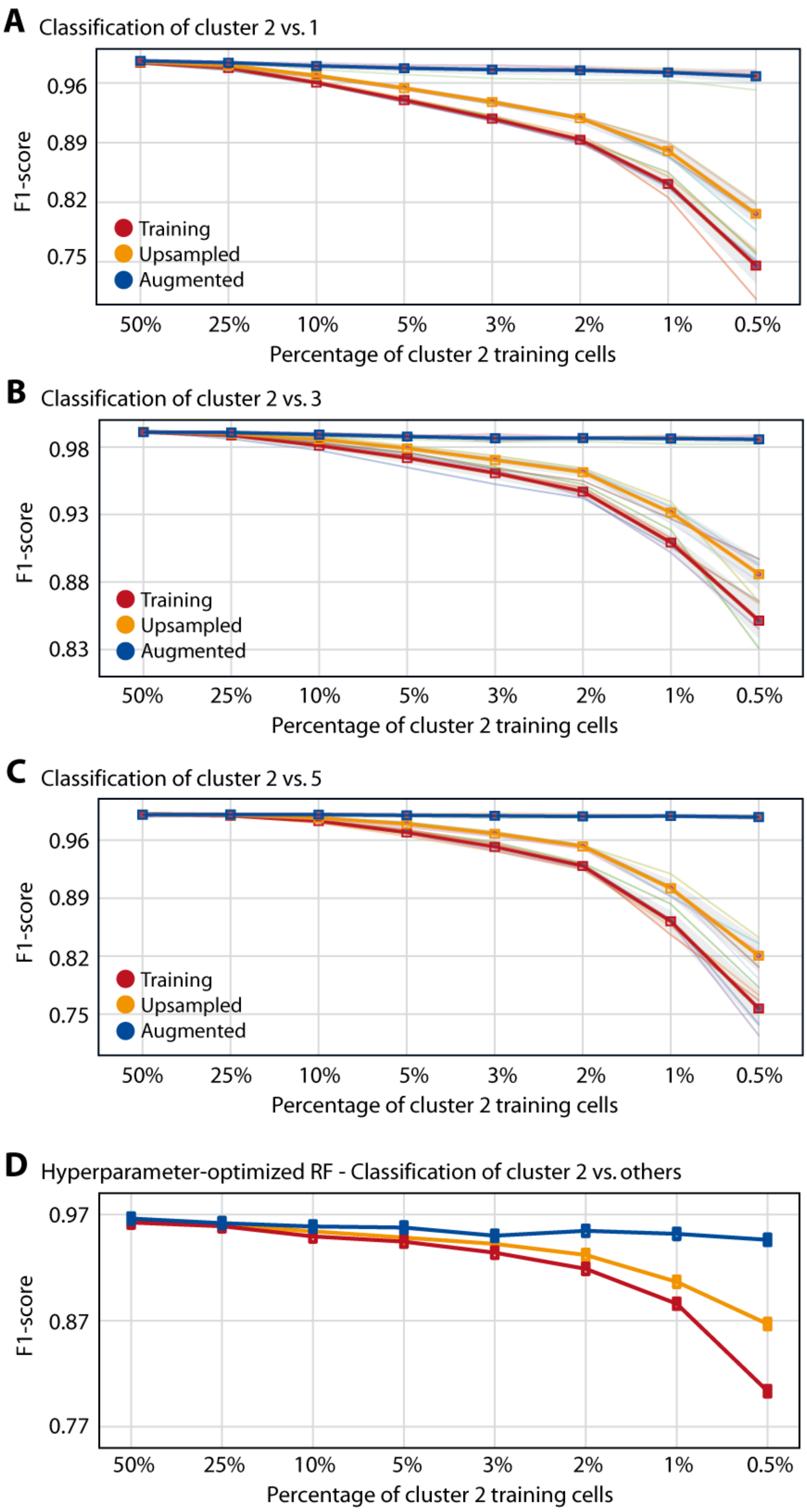

Supplementary Figure 14 *RF classification performance for three cluster-specific comparisons.* F1-score reached by an RF classifier trained to discriminate (A) cluster 2 from cluster 1, (B) cluster 2 from cluster 3, and (C) cluster 2 from cluster 5 cells. D shows the F1-score reached by a hyper-parameter-optimized RF trained to discriminate cluster 2 from other

cells. The RFs were trained on training (red), upsampled (yellow), or augmented (blue) datasets for eight different levels of downsampling (50% to 0.5%). Panels A-C show the mean F1-score over 5 different samplings of the training sets (individual folds in gray) whereas panel D shows the F1-score for a single training set initialization.

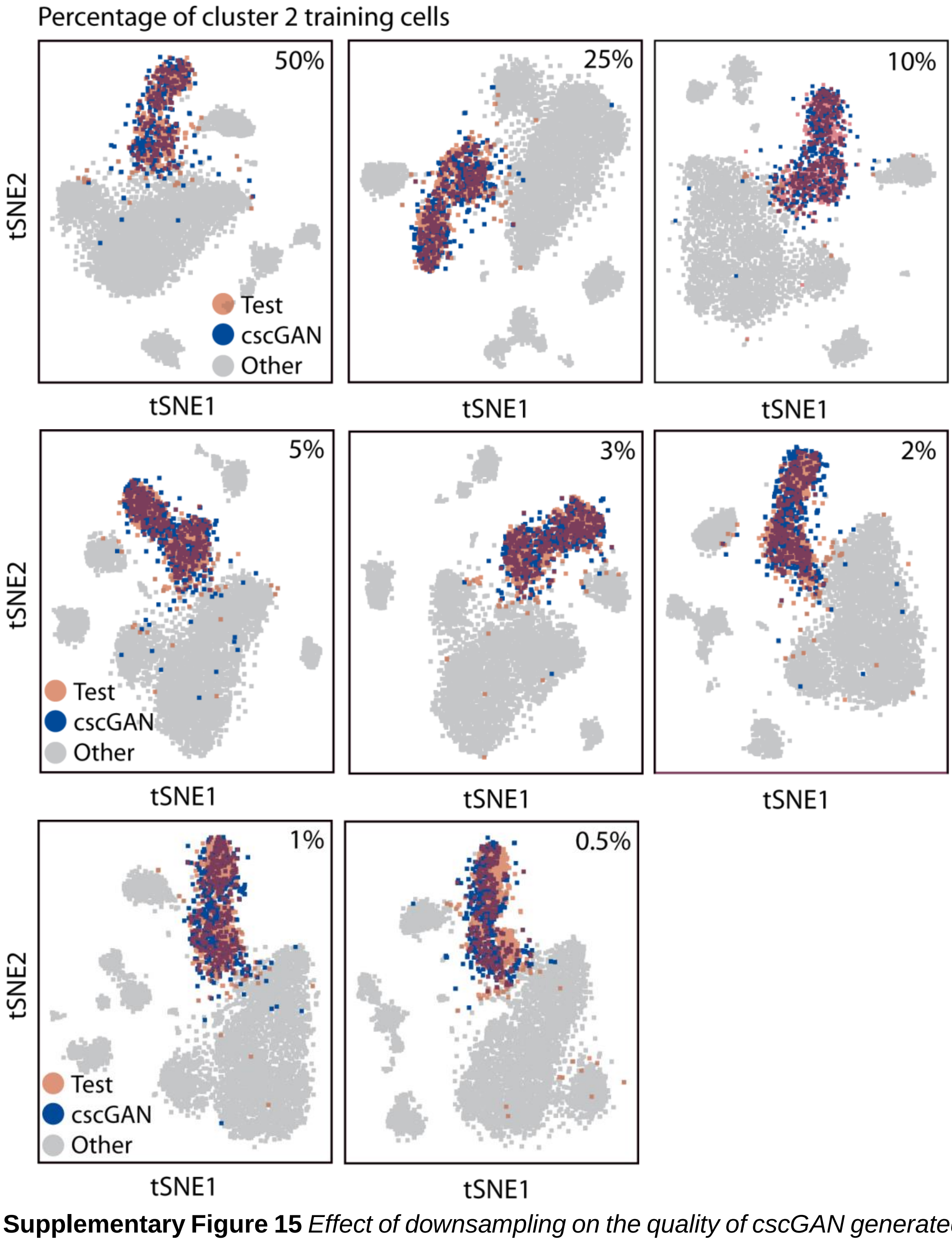

Supplementary Figure 15 *Effect of downsampling on the quality of cscGAN generated cluster 2 cells.* Each subfigure is a t-SNE representation of real test (red) and cscGAN generated (blue) cluster 2 cells for different levels of downsampling (50% to 0.5%). Gray cells represent real test data of other clusters.

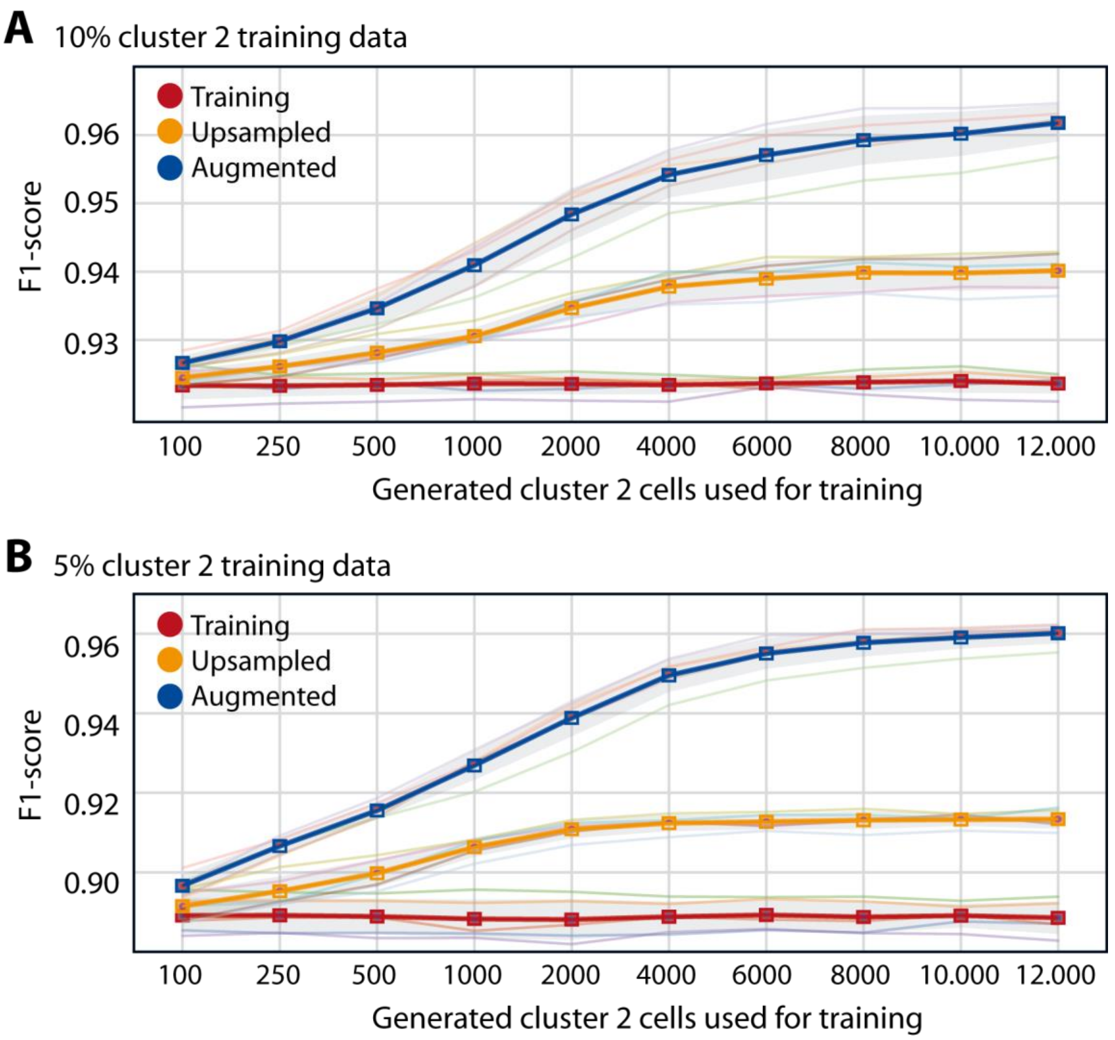

Supplementary Figure 16 *RF classification performance for different numbers of cluster 2 cells.* F1-score reached by an RF classifier discriminating cluster 2 from other cells when trained on (A) 10% or (B) 5% downsampled (red), upsampled (yellow), and augmented (blue) cells. The different numbers of upsampled and augmented cells used for training are shown on the x-axis. It is important to note that the number of cluster 2 training cells did not change for the red curve. The results represent the mean for five different data partitions (seeds).

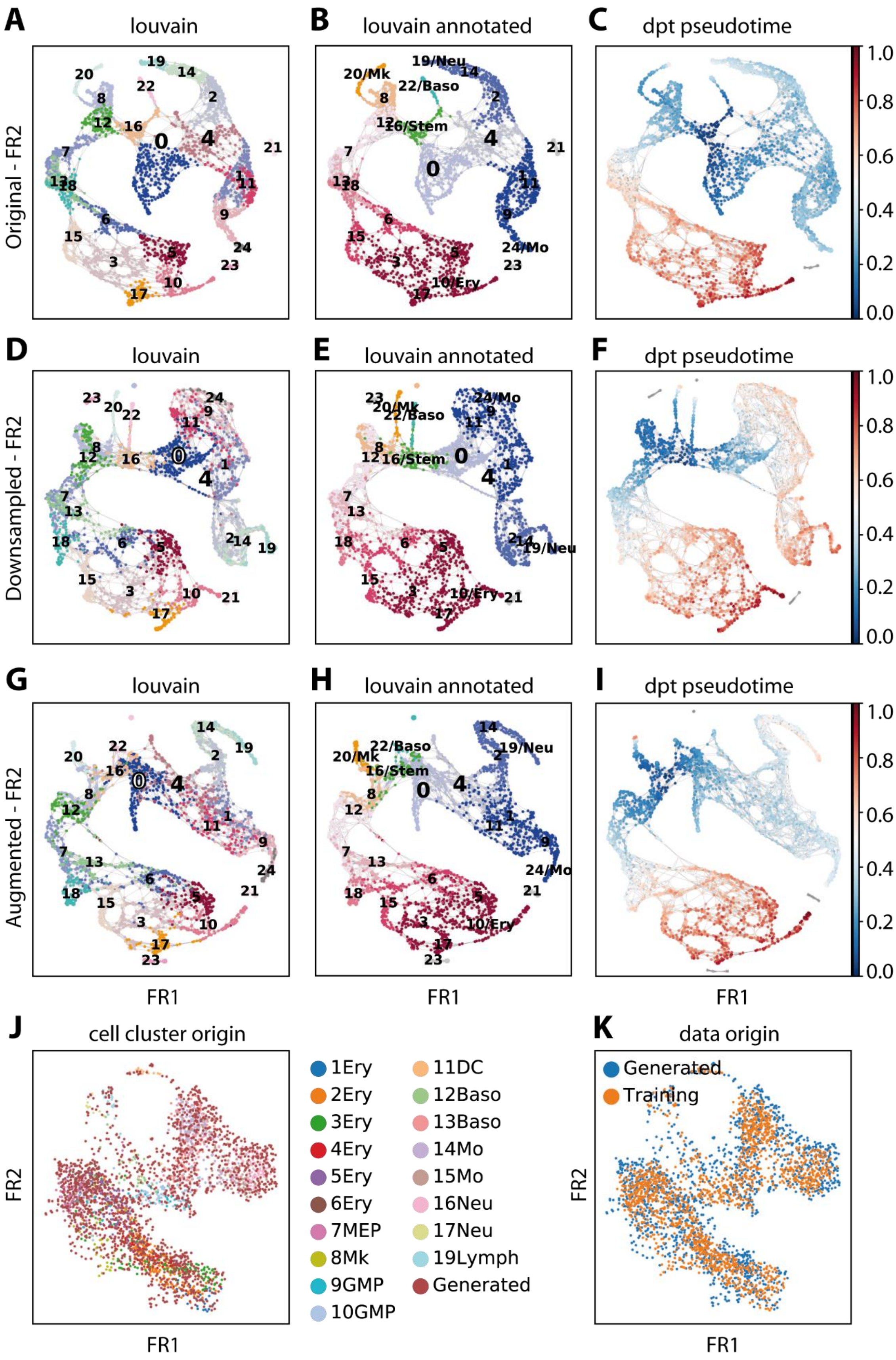

Supplementary Figure 17 *Force-directed graph visualizations of the Bone Marrow dataset.*

A-C: force-directed graphs of the original Bone Marrow dataset with Louvain groups (panel A),

manually annotated Louvain groups (panel B) and pseudo-time inferred by PAGA (panel C). D-F: force-directed graphs of the downsampled Bone Marrow dataset (cluster 4 is downsampled from the original dataset) with Louvain groups (panel D), manually annotated Louvain groups (panel E) and pseudo-time inferred by PAGA (panel F). The downsampling is affecting the structure of the graph around clusters 0, 1, 4 and 11. G-I: force-directed graphs of the augmented Bone Marrow dataset (cluster 4 is downsampled from the original dataset then augmented using cells generated by scGAN trained on the downsampled dataset) with Louvain groups (panel G), manually annotated Louvain groups (panel H) and pseudo-time inferred by PAGA (panel I). The structure of the original graph is restored. J-K: force-directed graphs of the real and scGAN-generated cells with their Louvain clustering (panel J) and their origin (panel K).

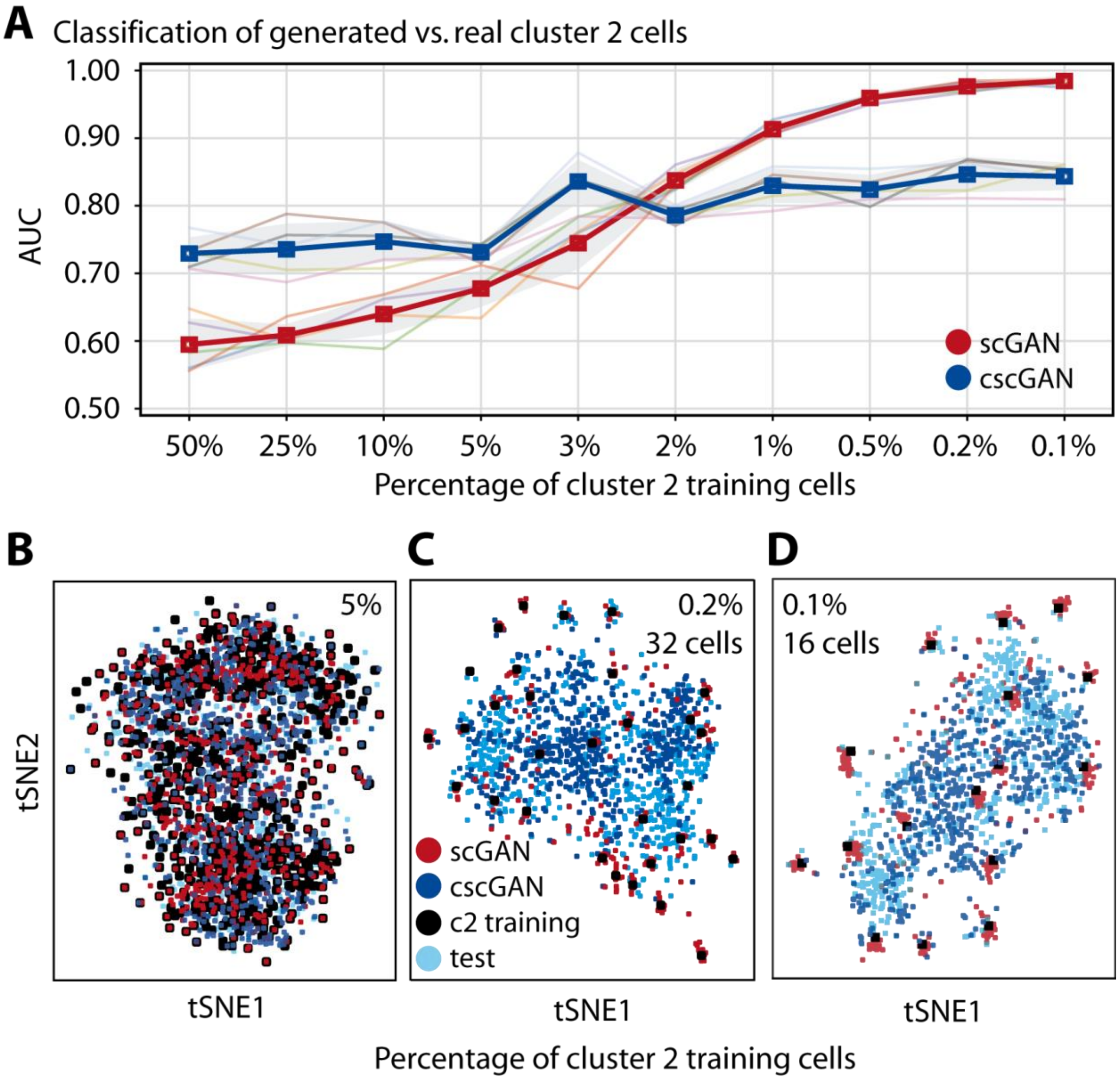

Supplementary Figure 18 *RF classification and t-SNE visualization for scGAN and cscGAN models trained on cluster 2 PBMC cells.* scGAN models were trained only on different amounts of cluster 2 cells (50% to 0.1% of all cluster 2 cells), whereas cscGAN models were trained on the same number of cluster 2 cells and all other cell types. A: AUC of an RF classifier trained to classify cluster 2 real from generated cells (cluster 2 specific scGAN in red, cscGAN in blue). B-D: t-SNE visualization of cluster 2 (cluster 2 specific) scGAN generated cells (red), cscGAN generated cells (blue), real training cells (black) and real test cells (light blue), for different levels of downsampling of the cluster 2 training cells (5% for panel B, 0.2% for the panel C and 0.1% for panel D).
