# Supplementary material for: Realistic in silico generation and augmentation of single-cell RNA-seq data using generative adversarial networks
Source: Nat Commun. 2020 Jan 9;11:166. doi: 10.1038/s41467-019-14018-z (PMC6952370; doi:10.1038/s41467-019-14018-z)
Supplement: Supplementary file 3 — Reporting Summary [file 41467_2019_14018_MOESM3_ESM.pdf]

natureresearch

Corresponding author(s): Stefan Bonn

Last updated by author(s): Dec 13, 2019

Reporting Summary

Nature Research wishes to improve the reproducibility of the work that we publish. This form provides structure for consistency and transparency in reporting. For further information on Nature Research policies, see Authors & Referees and the Editorial Policy Checklist.

Statistics

For all statistical analyses, confirm that the following items are present in the figure legend, table legend, main text, or Methods section.

n/a	Confirmed	
☐	☒	The exact sample size (*n*) for each experimental group/condition, given as a discrete number and unit of measurement
☐	☒	A statement on whether measurements were taken from distinct samples or whether the same sample was measured repeatedly
☐	☒	The statistical test(s) used AND whether they are one- or two-sided<br>*Only common tests should be described solely by name; describe more complex techniques in the Methods section.*
☐	☒	A description of all covariates tested
☐	☒	A description of any assumptions or corrections, such as tests of normality and adjustment for multiple comparisons
☐	☒	A full description of the statistical parameters including central tendency (e.g. means) or other basic estimates (e.g. regression coefficient) AND variation (e.g. standard deviation) or associated estimates of uncertainty (e.g. confidence intervals)
☐	☒	For null hypothesis testing, the test statistic (e.g. *F*, *t*, *r*) with confidence intervals, effect sizes, degrees of freedom and *P* value noted<br>*Give P values as exact values whenever suitable.*
☒	☐	For Bayesian analysis, information on the choice of priors and Markov chain Monte Carlo settings
☒	☐	For hierarchical and complex designs, identification of the appropriate level for tests and full reporting of outcomes
☐	☒	Estimates of effect sizes (e.g. Cohen's *d*, Pearson's *r*), indicating how they were calculated

Our web collection on statistics for biologists contains articles on many of the points above.

Software and code

Policy information about availability of computer code

Data collection	We downloaded data from the sources mentioned in the manuscript.
Data analysis	Our (c)scGAN Tensorflow49 implementation can be found on https://github.com/imsb-uke/scGAN, including documentation for the training of the (c)scGAN models. As mentioned before, we used Scanpy32 to conduct most of the data analysis. We also compared our results to those of Splatter22, and adapted the code they provided on Github (https://github.com/Oshlack/splatter).<br>For the sake of reproducibility, here is a list of the version of all the packages we used: Tensorflow v1.8, Scanpy v1.2.2, Anndata v0.6.5, Pandas v0.22.0, Numpy v1.14.3, Scipy v1.1.0, Scikit-learn v0.19.1, R v3.5.0 (2018-04-23), loomR v0.2.0, SHOGUN v6.1.3, SingleCellexperiment v1.2.0, Splatter v1.4.0, SUGAR v0.0, MAGIC v1.3.0, SCENIC v0.1.7, GENIE3 v1.0.0, Rcistarget v0.99.0, AUCell v0.99.5, RcisTarget.mm9.motifDatabases.20k v0.1.1, ZIFA v0.1.Regarding hardware, all (c)scGAN models were trained on a single-GPU of an NVIDIA DGX-1 server (Tesla V100 GPUs).

For manuscripts utilizing custom algorithms or software that are central to the research but not yet described in published literature, software must be made available to editors/reviewers. We strongly encourage code deposition in a community repository (e.g. GitHub). See the Nature Research guidelines for submitting code & software for further information.

Data

Policy information about availability of data

All manuscripts must include a data availability statement. This statement should provide the following information, where applicable:

- Accession codes, unique identifiers, or web links for publicly available datasets
- A list of figures that have associated raw data
- A description of any restrictions on data availability

The datasets used and analysed during the current study are available on the 10x Genomics dataset repository at https://support.10xgenomics.com/single-cell-gene-expression/datasets/1.1.0/fresh_68k_pbmc_donor_a for PBMC, at https://support.10xgenomics.com/single-cell-gene-expression/datasets/1.3.0/1M_neurons

for Brain small and Brain large, and in the Gene Expression Omnibus repository, at https://www.ncbi.nlm.nih.gov/geo/query/acc.cgi?acc=GSE72857 for Bone Marrow and https://www.ncbi.nlm.nih.gov/geo/query/acc.cgi?acc=GSE60361 for Zeisel.

Field-specific reporting

Please select the one below that is the best fit for your research. If you are not sure, read the appropriate sections before making your selection.

☒ Life sciences ☐ Behavioural & social sciences ☐ Ecological, evolutionary & environmental sciences

For a reference copy of the document with all sections, see nature.com/documents/nr-reporting-summary-flat.pdf

Life sciences study design

All studies must disclose on these points even when the disclosure is negative.

Sample size	Not applicable since we only downloaded published data.
Data exclusions	We did not excluded data.
Replication	We used several different datasets and a training/cross validation/testing approach to guarantee model generalization.
Randomization	Random sampling was performed keeping the general composition of the sample close to that of the complete dataset with regard to covariates using the scikitlearn python library (balanced sampling).
Blinding	Not applicable (see above).

Reporting for specific materials, systems and methods

We require information from authors about some types of materials, experimental systems and methods used in many studies. Here, indicate whether each material, system or method listed is relevant to your study. If you are not sure if a list item applies to your research, read the appropriate section before selecting a response.

Materials & experimental systems

n/a	Involved in the study	
☒	☐	Antibodies
☒	☐	Eukaryotic cell lines
☒	☐	Palaeontology
☒	☐	Animals and other organisms
☒	☐	Human research participants
☒	☐	Clinical data

Methods

n/a	Involved in the study	
☒	☐	ChIP-seq
☒	☐	Flow cytometry
☒	☐	MRI-based neuroimaging
